# Prevalence and characteristics of visual snow syndrome in a cohort of young Italian adults

**DOI:** 10.1111/ene.16472

**Published:** 2024-09-24

**Authors:** Marina Romozzi, Vincenzo Trigila, Federico Tosto, Giovanni Cuffaro, David García‐Azorín, Luigi Francesco Iannone, Pietro Romozzi, Gustavo Savino, Paolo Calabresi, Francesca Puledda, Catello Vollono

**Affiliations:** ^1^ Dipartimento Universitario di Neuroscienze Università Cattolica del Sacro Cuore Rome Italy; ^2^ Neurologia, Dipartimento di Neuroscienze, Organi di Senso e Torace, Fondazione Policlinico Universitario A. Gemelli IRCCS Rome Italy; ^3^ Department of Neuroscience “Giovanni Paolo II” Hospital Lamezia Terme Italy; ^4^ Oculistica, Fondazione Policlinico Universitario A. Gemelli IRCCS Rome Italy; ^5^ Hospital Universitario Rio Hortega Valladolid Spain; ^6^ Department of Medicine, Toxicology and Dermatology, Faculty of Medicine University of Valladolid Valladolid Spain; ^7^ Section of Clinical Pharmacology and Oncology, Department of Health Sciences University of Florence Florence Italy; ^8^ Dipartimento di Scienze Farmaceutiche Università degli Studi di Perugia Perugia Italy; ^9^ Headache Group, Wolfson SPaRRC, Institute of Psychiatry, Psychology and Neuroscience King's College London London UK

**Keywords:** migraine, palinopsia, prevalence, tinnitus, visual snow

## Abstract

**Background:**

Visual snow (VS) and visual snow syndrome (VSS) are becoming increasingly recognized. However, their prevalence worldwide is unknown. This study aimed to investigate lifetime prevalence and describe the clinical characteristics of VS and VSS in a representative population sample from Italy.

**Methods:**

This cross‐sectional study was conducted among students attending different faculties in three universities in the central and southern regions of Italy. Eligible participants completed a self‐administered questionnaire. In patients fulfilling possible criteria for VS/VSS, the diagnosis was validated by an on‐site visit conducted by experienced neurologists and neuro‐ophthalmologists that included optical coherence tomography angiography (OCTA).

**Results:**

A total of 750 participants completed the study. Seven (0.9%) reported symptoms compatible with VS (mean age 24.8 ± 3.85 years). Among the seven patients, five (0.7%) also met the phenomenological and temporal criteria for VSS. Neuroimaging and ophthalmological examinations showed normal results upon review or during the on‐site visit including OCTA. For the five patients with full VSS, the other visual symptoms reported were enhanced entoptic phenomenon (*n* = 5), photophobia (*n* = 5), palinopsia (*n* = 1), and nyctalopia (*n* = 4). In four of the seven patients (57%) reporting VS symptoms, there was a concomitant diagnosis of migraine with aura, and in one (14%) migraine without aura. All patients (*n* = 7) reported tinnitus. Six of the seven (85.7%) patients with VS/VSS had never used specific treatments for the condition. None of the seven patients had received a previous diagnosis of VS/VSS.

**Conclusions:**

The prevalence in Italy of VSS is around 1%. However, there is a limited tendency for affected individuals to seek medical attention, leading to a low rate of diagnosis and treatment.

## INTRODUCTION

Visual snow (VS) is an alteration in visual perception described as persistent innumerable dots over the entire visual field, often compared with television static [1, 2]. In addition to the VS phenomenon, patients often report other visual disturbances such as palinopsia, entoptic phenomena, nyctalopia, and photophobia, constituting the visual snow syndrome (VSS) [1]. Migraine and tinnitus are often comorbid with VSS, reported in up to 75% of patients [2].

The pathogenesis of VSS has not yet been clarified. It has been proposed to be a network disorder involving changes in cortical excitability within thalamocortical circuits, alongside alteration in several neurotransmitters like glutamate and serotonin [3, 4, 5].

Since its initial description in 1995 and the proposal of specific criteria in 2014, VSS has increasingly been recognized as a distinct clinical entity [1, 6]. However, VS and VSS are still overlooked, and data on their prevalence are lacking. To date, only one study has examined the prevalence of these conditions in the United Kingdom (UK) population, reported as 3.7% for VS and 2.2% for VSS [7]. No studies have evaluated the prevalence of VS and VSS in Italy.

This study aimed to investigate the point prevalence and describe the clinical characteristics of VS and VSS in a cohort of Italian young adults.

## METHODS

This cross‐sectional study was conducted among students attending different faculties in three universities located in the central and southern regions of Italy (Università Cattolica del Sacro Cuore in Rome, Università degli studi Magna Græcia in Catanzaro, and Università degli Studi di Perugia in Perugia) from July 2023 to March 2024. Eligible participants were 18 years or older and completed a self‐administered questionnaire designed following the criteria proposed by Schankin and colleagues [1].

The questionnaire was thoroughly reviewed by two board‐certified neurologists and headache specialists (M.R. and C.V.).

Participants in the study were treated following the principles of the Helsinki Declaration of Biomedical Ethics. The study was approved by the Ethical Committee of the Università Cattolica del Sacro Cuore (protocol ID4155/2021).

Before data collection, all participants provided written informed consent and provided consent to be contacted for a telemedicine or in‐person visit. Personal information collected from the questionnaires was anonymized.

The questionnaire was in Italian and comprised several sections. In the first section, basic demographic information, including age, gender, and family history of migraine, were collected. The second section displayed five images illustrating the VS phenomenon, sourced from published papers and the VS initiative website (https://www.visualsnowinitiative.org) (Figure S1) [1]. In this section, participants were questioned whether they had experienced the phenomenon represented in the images, characterized by dynamic, continuous, minuscule dots across the entire visual field.

If the participant responded affirmatively, they were asked whether this experience lasted more than 3 months. The second section collected data on the associated symptoms of VSS. Specifically, it examined the presence of palinopsia (which was described and illustrated in pictures as after images or trailing) and its association with enhanced entoptic phenomena (which were described as excessive floaters in both eyes, excessive blue field entoptic phenomenon, self‐light of the eye, or spontaneous photopsia) (Figure S1). Additionally, the section assessed the presence of photophobia and nyctalopia.

The final section of the questionnaire focused on other medical conditions, particularly the concomitant presence of migraine and tinnitus and chronic treatments for migraine or other disturbances. Finally, participants were asked if they had previously been diagnosed with VS or VSS or if they had used any treatment for the condition in the past.

Replies to the questionnaire were screened by a fully trained neurologist (M.R., C.V., or F.T.). Patients who responded affirmatively to the second section and thus reported the VS phenomenon, as well as those with additional symptoms indicative of VSS, were contacted for a telemedicine visit.

During the telemedicine visit, a neurologist confirmed VS/VSS symptoms, duration, age of onset, characteristics, previous diagnosis of VS/VSS, and any previous treatment. The concomitant diagnosis of migraine was also confirmed when present, according to International Classification of Headache Disorders, 3rd Edition (ICHD‐3) criteria [8]. Neuroimaging investigations previously conducted were also examined. Finally, previous ophthalmological evaluations were reviewed by a board‐certified neuro‐ophthalmologist (G.C.).

Patients who had not undergone an ophthalmological evaluation, including optical coherence tomography (OCT), were invited for an on‐site visit. Brain magnetic resonance imaging (MRI) was prescribed to patients who had not undergone one previously.

The on‐site visit included an ophthalmological evaluation conducted by a neuro‐ophthalmologist, including measurement of ocular pressure, ocular motility, best‐corrected visual acuity, fundoscopy, and optical coherence tomography angiography (OCTA) with the Solix Fullrange OCT (Version 2019 V1.0.0.305; Optovue Inc., Freemont, CA, USA), and spectral domain‐OCTA, to exclude concomitant ocular pathologies.

Statistical data were analyzed using SPSS Version 21.0. Numerical variables were presented as mean and standard deviation and categorical variables as absolute numbers (*n*) and percentages (%).

## RESULTS

The survey was distributed among 800 patients attending the three universities. A total of 750 subjects (93.7%) completed the study and fulfilled the eligibility criteria, with a mean age (± standard deviation) of 23.2 ± 4.5 years, and 478 subjects were female (63.7%).

**FIGURE 1:**
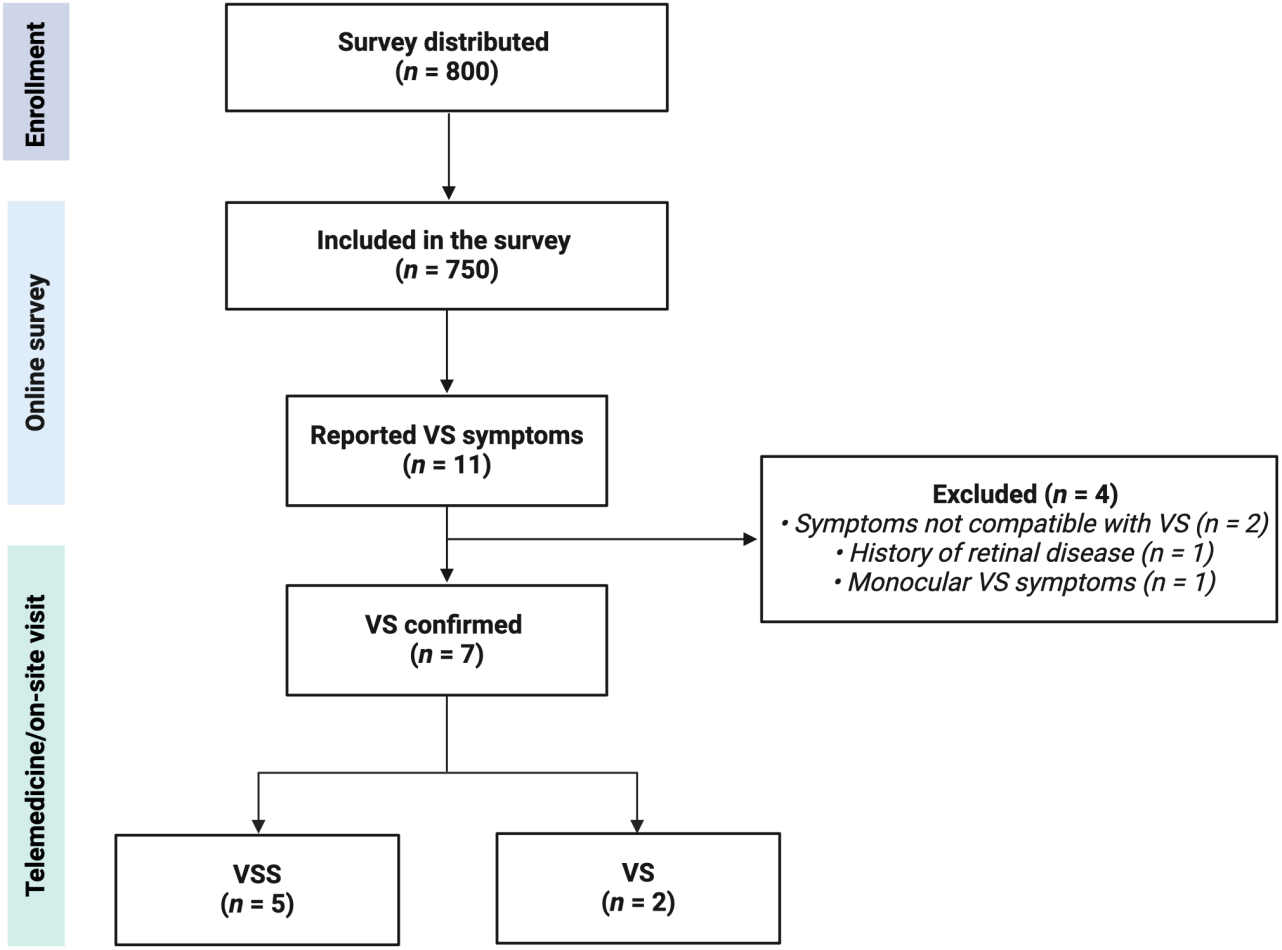
Flowchart of the study. VS, visual snow; VSS, visual snow syndrome.

A total of 11 participants (3.7%) reported symptoms compatible with VS during their lifetime. All underwent a telemedicine visit. Subsequently, four participants (0.5%) were excluded; two reported visual symptoms that were not consistent with the VS phenomenon (compatible with transient floaters that occur occasionally and last for a few seconds), one patient experienced disturbance exclusively in one eye despite a normal ophthalmological examination, OCT, and brain MRI scan, and one patient had a previous diagnosis of idiopathic maculopathy that was confirmed during the on‐site visit. Figure 1 shows the study flowchart, and Table 1 summarizes the detailed characteristics of the seven included VS patients, who had a mean age of 24.8 ± 3.85 years.

**TABLE 1:** Detailed clinical characteristics of patients reporting visual snow symptoms.

Subject	Age (y)/sex	VS age of onset	VS duration	VS static type	VSS diagnosis	Palinopsia	Enhanced entoptic phenomenon	Photophobia	Nyctalopia	Comorbid migraine	Tinnitus	Brain MRI	Ophthalmological evaluation including OCT
#1	25/F	24	8 m	Coloured	Yes	−	+	+	+	MwA	+	Normal	Normal
#2	23/F	22	1 y	N/A	Yes	−	+	+	+	−	+	Normal	Normal
#3	23/F	6	>10 y	Coloured	Yes	+	+	+	−	MwA	+	Normal	Normal
#4	33/M	32	9 m	White	No	−	+	−	−	MwA	+	Normal	Normal
#5	23/F	22	12 m	White or coloured	No	−	−	−	+	MwA	+	Normal	VF: loss in left superior quadrant^a^ OCT: normal
#6	23/F	N/A	>3 m	N/A	Yes	−	+	+	+	MwoA	+	Normal	Normal
#7	21/F	21	3 m	N/A	Yes	−	+	+	+	−	+	Normal	Normal

Abbreviations: F, female; M, male; m, months; MRI, magnetic resonance imaging; MwA, migraine with aura; MwoA, migraine without aura; N/A, not available; OCT, optical coherence tomography; VF, visual field; VS, visual snow; VSS, visual snow syndrome; y, years; +, present; −, absent.

^a^ The visual field deficits were interpreted as functional by neuro‐ophthalmologists.

Among the seven patients (0.9%) reporting VS symptoms, five (0.7%) also met the phenomenological and temporal criteria for VSS (i.e., at least two additional visual symptoms and all symptoms persisting for over 3 months). No patient reported prior use of illicit drugs. All neuroimaging and ophthalmological examinations showed normal results upon review or during the on‐site visit, including OCT and OCTA.

For the five patients with full VSS, the other visual symptoms reported were enhanced entoptic phenomenon (*n* = 5), photophobia (*n* = 5), palinopsia (*n* = 1), and nyctalopia (*n* = 4). In four patients (57%) of the seven reporting VS symptoms, there was a concomitant diagnosis of migraine with aura as per the ICHD‐3 criteria, and in one patient (14%) of migraine without aura, and all patients (*n* = 7) reported tinnitus.

Six of the seven patients with VS/VSS had never used specific treatments for the condition. One had used several preventive treatments, including pregabalin, vortioxetine, bupropion, and alprazolam, reporting no effectiveness or change in symptoms. None of the seven patients had received a previous formal diagnosis of VS/VSS.

## DISCUSSION

The prevalence of VS among our cohort of Italian young adults was 0.9% and 0.7% for VSS. The patients reporting VS symptoms had a mean age of 24.8 ± 3.85 years and were predominantly female (86%).

Additionally, 72% of the subjects had a concomitant diagnosis of migraine and 57% of migraine with aura; all patients reported tinnitus.

Worldwide, data on the prevalence of VS are lacking. To date, only one epidemiological study, based on an online survey, has assessed the prevalence of VS and VSS in the UK population [7]. The authors found a prevalence of 3.7% for VS and 2.2% for VSS in a cohort of 1015 participants. In the study, the survey participants' reports were not validated by personal interviews [7]. Patients with symptoms compatible with VSS had a mean age of 50.6 ± 14 years and were mostly female (ratio of 1.6:1); 54.5% reported having headache, and 22.7% had migraine with visual aura; 59.1% of the patients reported tinnitus [7].

The prevalence of VS and VSS in our cohort was lower; importantly, however, our sample comprised volunteers within a specific age range that is not representative of the wider population.

Our results align with previous work on the comorbidity of migraine and tinnitus [7]. Puledda et al. recently described the clinical characteristics of 1100 patients with VS and VSS [2]. This cohort had no significant difference in the male‐to‐female ratio, and the mean age was approximately 29 years, with a mean age at the onset of the syndrome in childhood (12.8 ± 13.2 years). In the VSS group, 37% of the patients had migraine with aura and approximately 75% of the patients had comorbid migraine and tinnitus [2]. In a study conducted by Viana et al., which compared 100 patients with VSS from the UK and 100 from Italy, no significant clinical or demographic differences were found between the two groups. The Italian cohort had an average age of onset of 22 ± 10 years and, notably, 50% of these patients experienced a sudden onset of VSS [9].

The strong association between VS/VSS, migraine, and tinnitus suggests that these two comorbid conditions might share common pathophysiological mechanisms with VS.

This hypothesis was also corroborated by a study on 17 patients with VSS that investigated brain metabolism through [18F]‐fluorodeoxyglucose‐positron emission tomography. This study showed hypermetabolism of the right lingual gyrus in VSS, an area that is also involved in photophobia in migraine, possibly suggesting a dysfunctional cortical mechanism in both disorders [10, 11].

Tinnitus is a common disorder in adults, present in up to a quarter of the general population, and is defined as an auditory sensation characterized by perceiving sound without any external auditory stimulus [12].

In patients with VSS, the prevalence of tinnitus reaches 75% [2]. Theoretically, both VSS and tinnitus are different manifestations of a similar process, indicating underlying central mechanisms that may involve aberrant sensory processing within the association cortices or the thalamocortical network [2, 13, 14].

One interesting finding from our study, which was the first to assess the prevalence of VS/VSS in an Italian population, is that none of the seven patients with VS/VSS had received a prior diagnosis, indicating a general lack of awareness regarding this condition.

A strength of our study is that, unlike prior studies on VS prevalence, our survey responses were validated through both telemedicine and on‐site visits. These visits included comprehensive ophthalmological examinations to ensure the exclusion of secondary causes of the disturbances.

However, limitations should be acknowledged. As this was a survey‐based study, complex clinical concepts may not have been completely captured, potentially leading to underestimating the disorder, and the sample size of patients reporting VS/VSS was too small to draw definitive conclusions about the specific pattern of VSS symptoms reported. Moreover, by targeting students, a portion of the population could possibly have been excluded from the survey, therefore not fully representing the actual prevalence of these conditions, since participants could develop VSS during the remainder of their lifespan.

## CONCLUSIONS

VS and VSS affect almost 1% of the population, yet they remain poorly recognized, with an important lack of awareness surrounding these conditions.

## AUTHOR CONTRIBUTIONS

**Marina Romozzi:** Conceptualization; investigation; funding acquisition; writing – original draft; methodology; validation; visualization; writing – review and editing; data curation. **Vincenzo Trigila:** Investigation; methodology; data curation. **Federico Tosto:** Data curation; investigation; methodology. **Giovanni Cuffaro:** Investigation. **David García‐Azorín:** Visualization; writing – review and editing; writing – original draft; supervision. **Luigi Francesco Iannone:** Writing – original draft; supervision. **Pietro Romozzi:** Investigation. **Gustavo Savino:** Conceptualization. **Paolo Calabresi:** Supervision. **Francesca Puledda:** Supervision; writing – original draft; writing – review and editing. **Catello Vollono:** Resources; supervision; validation; methodology; investigation; writing – original draft.

## FUNDING INFORMATION

This research received no specific grants from funding agencies in the public, commercial, or not‐for‐profit sectors.

## CONFLICT OF INTEREST STATEMENT

The authors report no disclosures relevant to the article.

## Supporting information


Figure S1.

## Data Availability

The data that support the findings of this study are available from the corresponding author upon reasonable request.
