# Supplementary figures and images for: Prevalence and characteristics of visual snow syndrome in a cohort of young Italian adults
Source: Eur J Neurol. 2024 Sep 24;31(12):e16472. doi: 10.1111/ene.16472 (PMC11555012; doi:10.1111/ene.16472)

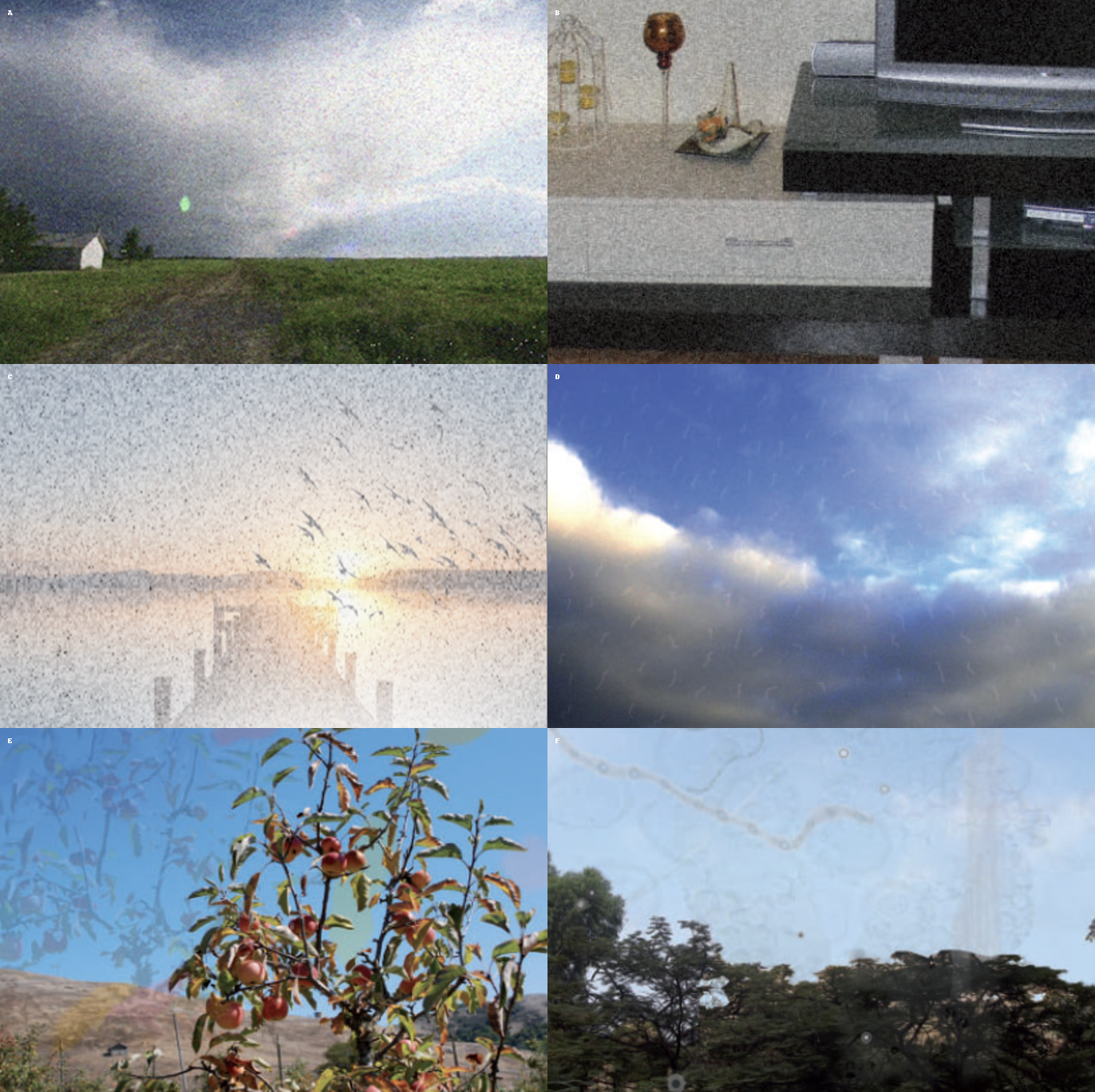

Supplement: Supplementary file 1 — Figure S1. [file ENE-31-e16472-s001.png]
